# Evaluating the Effects of Subnormothermic Perfusion with AP39 in a Novel Blood-Free Model of Ex Vivo Kidney Preservation and Reperfusion

**DOI:** 10.3390/ijms22137180

**Published:** 2021-07-02

**Authors:** Smriti Juriasingani, Ashley Jackson, Max Yulin Zhang, Aushanth Ruthirakanthan, George J. Dugbartey, Emrullah Sogutdelen, Max Levine, Moaath Mandurah, Matthew Whiteman, Patrick Luke, Alp Sener

**Affiliations:** 1 Department of Microbiology and Immunology, Western University, London, ON N6A 5C1, Canada; sjuriasi@uwo.ca (S.J.); yzha493@uwo.ca (M.Y.Z.); 2 Matthew Mailing Center for Translational Transplant Studies, London Health Sciences Centre, London, ON N6A 5A5, Canada; ajacks85@uwo.ca (A.J.); aruthira@uwo.ca (A.R.); profduu@yahoo.com (G.J.D.); max.levine@lhsc.on.ca (M.L.); moaath.mandurah@lhsc.on.ca (M.M.); patrick.luke@lhsc.on.ca (P.L.); 3 Department of Pathology & Laboratory Medicine, Western University, London, ON N6A 5C1, Canada; 4 Multi-organ Transplant Program, London Health Sciences Center, London, ON N6A 5A5, Canada; 5 Department of Pharmacology and Toxicology, School of Pharmacy, College of Health Sciences, University of Ghana, P.O. Box LG 43, Legon, Accra, Ghana; 6 Department of Urology, Bolu Abant Izzet Baysal University, Bolu 14030, Turkey; esdelen@gmail.com; 7 St. Luke’s Campus, University of Exeter Medical School, Exeter EX1 2HZ, UK; M.Whiteman@exeter.ac.uk

**Keywords:** kidney preservation, kidney transplantation, hydrogen sulfide (H_2_S), AP39, donation after cardiac death (DCD), subnormothermic

## Abstract

The use of blood for normothermic and subnormothermic kidney preservation hinders the translation of these approaches and promising therapeutics. This study evaluates whether adding hydrogen sulfide donor AP39 to Hemopure, a blood substitute, during subnormothermic perfusion improves kidney outcomes. After 30 min of renal pedicle clamping, porcine kidneys were treated to 4 h of static cold storage (SCS-4 °C) or subnormothermic perfusion at 21 °C with Hemopure (H-21 °C), Hemopure + 200 nM AP39 (H200nM-21 °C) or Hemopure + 1 µM AP39 (H1µM-21 °C). Then, kidneys were reperfused with Hemopure at 37 °C for 4 h with metabolic support. Perfusate composition, tissue oxygenation, urinalysis and histopathology were analyzed. During preservation, the H200nM-21 °C group exhibited significantly higher urine output than the other groups and significantly higher tissue oxygenation than the H1µM-21 °C group at 1 h and 2h. During reperfusion, the H200nM-21 °C group exhibited significantly higher urine output and lower urine protein than the other groups. Additionally, the H200nM-21 °C group exhibited higher perfusate pO_2_ levels than the other groups and significantly lower apoptotic injury than the H-21 °C and the H1µM-21 °C groups. Thus, subnormothermic perfusion at 21 °C with Hemopure + 200 nM AP39 improves renal outcomes. Additionally, our novel blood-free model of ex vivo kidney preservation and reperfusion could be useful for studying other therapeutics.

## 1. Introduction

Kidney transplantation is the preferred treatment for end-stage kidney disease (ESKD) because it improves long-term survival and quality of life compared to hemodialysis [1,2,3]. However, there is a critical shortage of donor kidneys across the globe due to the increasing need for kidney transplants. In Canada, the prevalence of ESKD has risen by 33% between 2010 and 2019 [4]. Despite the 1648 adult kidney transplants that were performed in 2019, 3261 individuals remained on the waiting list [5]. To meet the rising demand, kidneys from marginal donors are being used more frequently. While donation after brain death does not impact organ health, donation after cardiac death (DCD) leads to warm ischemic injury, as blood flow decreases and the heart stops. This is of concern because DCD kidneys are being transplanted more frequently and often lead to poorer patient outcomes compared to kidneys from other donors [6]. Additionally, clinical cold preservation methods (4 °C), such as static cold storage (SCS), exacerbate DCD kidney outcomes due to the combination of cold and warm ischemic injury [7,8,9].

Several strategies have been explored to improve cold preservation methods, such as the addition of hydrogen sulfide (H_2_S), a gasotransmitter with cytoprotective properties, to preservation solutions [10,11,12]. Our group and others have previously shown that adding nanomolar concentrations of the H_2_S donor, AP39, to cold preservation solution improves renal and cardiac graft outcomes [11,13]. Additionally, there is growing interest in alternatives to cold preservation that employ normothermic (36–37 °C) and subnormothermic (15–35 °C) temperatures [14]. Several porcine and discarded human kidney studies have shown that normothermic and subnormothermic machine perfusion improve DCD renal graft outcomes compared to SCS on ice [15,16,17,18,19,20]. However, a major challenge in this context is the need for oxygenation and nutrients to meet the metabolic demand of the kidney at these higher temperatures. Previous studies have primarily used erythrocyte-based solutions or autologous whole blood for this purpose [16,18,21], but the use of blood is a challenge for the clinical translation of these approaches. Pervasive shortages of banked blood and the challenges associated with obtaining blood from DCD donors limit the availability of blood for ex vivo kidney preservation.

The use of blood substitutes, especially hemoglobin-based oxygen carriers (HBOC), can solve the problem of oxygenation and circumvent the need for blood in the context of normothermic and subnormothermic preservation. One promising candidate is Hemopure (also known as HBOC-201), which is made of highly purified bovine hemoglobin [22,23]. Clinically, Hemopure is used to treat anemia in patients who cannot receive blood transfusions [24,25]. However, several recent studies have shown that ex vivo kidney preservation with Hemopure can improve renal graft outcomes. In 2019, Aburawi et al. [26] reported that normothermic perfusion (37 °C) of discarded human kidneys with Hemopure vs. packed red blood cells led to comparable outcomes. Subsequently, a recent study by our center has shown that subnormothermic perfusion (22 °C) of DCD porcine kidneys with Hemopure vs. whole blood exhibited similar outcomes, which further confirms the feasibility of Hemopure for blood-free renal graft preservation [19].

While recent evidence supports the use of Hemopure for kidney preservation, its potential as a platform to deliver therapeutics that could further enhance renal graft outcomes remains unexplored. We have previously shown that adding H_2_S donor, AP39, to University of Wisconsin (UW) solution, a preservation solution that is commonly used for SCS, made it suitable for subnormothermic preservation [27]. Additionally, we have shown that subnormothermic perfusion (21 °C) of DCD porcine kidneys with AP39-supplemented whole blood improves renal graft outcomes and reduces tissue injury using an ex vivo model of blood-based preservation and reperfusion [20]. In this study, we investigate whether subnormothermic perfusion at 21 °C with AP39-supplemented Hemopure improves DCD porcine renal graft outcomes compared to SCS and subnormothermic perfusion with Hemopure alone. To evaluate this aim, we use a novel blood-free model of ex vivo preservation and reperfusion. In this model, after 4 h of blood-free preservation, all kidneys are reperfused (37 °C) for 4 h with Hemopure, supplemented with a diuretic and metabolic support, to mimic the post-transplant milieu.

## 2. Results

### 2.1. Subnormothermic Perfusion with Hemopure + 200 nM AP39 at 21 °C Improves Gross Kidney Morphology and Perfusate pO_2_ Levels during Blood-Free Reperfusion

**Figure 1:**
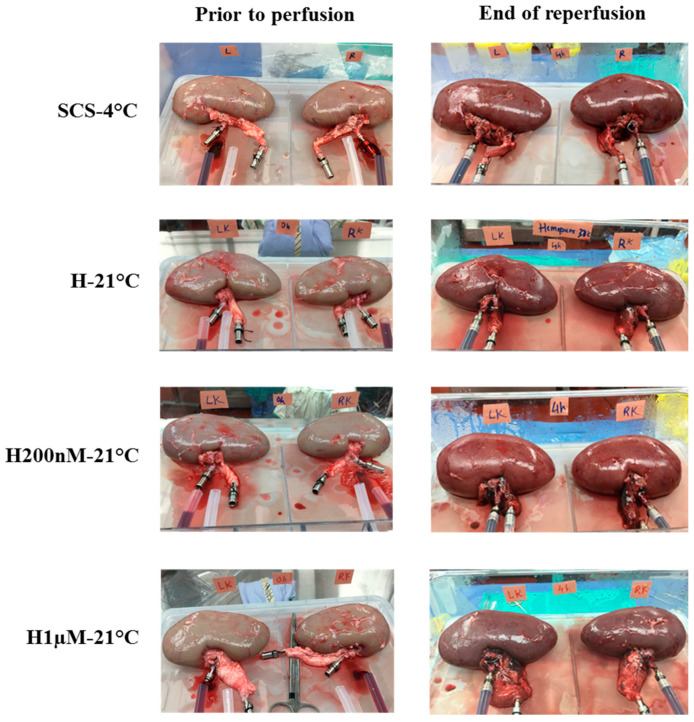
Gross morphology of the kidneys prior to preservation and at the end of reperfusion. Images were taken prior to perfusion to document the consistent flushing of the kidneys following the induction of warm ischemia. Images were taken at the end of reperfusion to document the gross morphology of kidneys by the end of the experiment. One pair of representative images were chosen for each preservation treatment group. Treatment groups: SCS-4 °C, static cold storage on ice at 4 °C. H-21 °C, perfusion with Hemopure at 21 °C. H200nM-21 °C, perfusion with Hemopure + 200 nM AP39 at 21 °C. H1µM-21 °C, perfusion with Hemopure + 1 µM AP39 at 21 °C.

**Figure 2:**
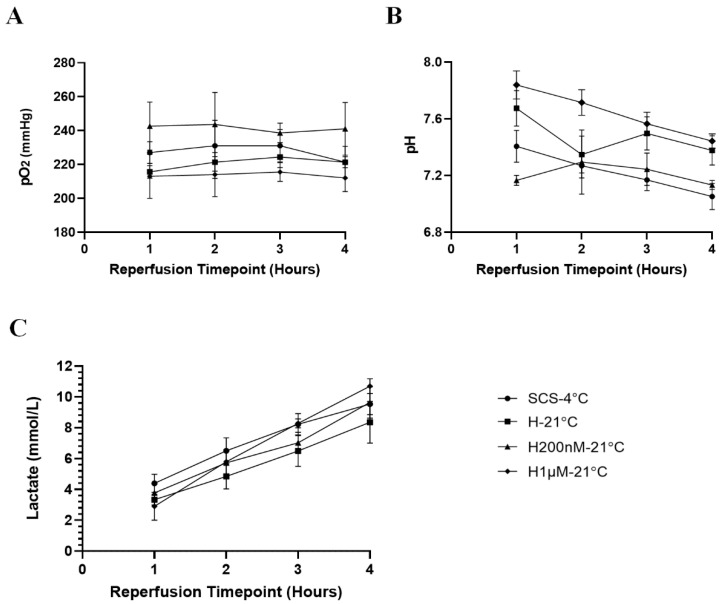
Perfusate parameters during blood-free reperfusion. (**A**) pO_2_ (mmHg), (**B**) pH and (**C**) Lactate (mmol/L) during blood-free reperfusion. Since pairs of kidneys were reperfused with shared perfusate, singular readings were obtained at each timepoint for each pair of kidneys. The iSTAT Analyzer was unable to detect tissue oxygenation for one pair of kidneys (*n* = 1) in the H1µM-21 °C group. Individual points on each graph represent the mean value ± SEM at a specific timepoint for pairs of kidneys within a single treatment group. Mean ± SEM values are listed in Table 1. After a Geisser–Greenhouse correction, values were compared using repeated measures two-way ANOVA followed by Tukey’s post-hoc test and no significant differences were found. Treatment groups: SCS-4 °C, static cold storage on ice at 4 °C (*n* = 3). H-21 °C, perfusion with Hemopure at 21 °C (*n* = 3). H200nM-21 °C, perfusion with Hemopure + 200 nM AP39 at 21 °C (*n* = 3). H1µM-21 °C, perfusion with Hemopure + 1 µM AP39 at 21 °C (*n* = 2).

Kidneys were imaged prior to preservation (after flushing) and at the end of reperfusion to evaluate differences in gross morphology (Figure 1). The images taken prior to preservation show that the kidneys in each group had similar gross morphology and were thoroughly flushed following the induction of warm ischemia, which is reflected by the pale colour. At the end of reperfusion, the H200nM-21 °C kidneys looked the reddest, which is an indicator of consistent perfusion and overall organ health. On the other hand, the H1µM-21 °C kidneys looked much darker than the other groups at the end of reperfusion, which is an indicator of thrombosis and tissue injury (Figure 1). Additionally, perfusate samples were analyzed hourly during reperfusion to evaluate relative changes in pO_2_, pH and lactate levels (Figure 2, Table 1). The H200nM-21 °C group exhibited higher perfusate pO_2_ levels than the SCS-4 °C, H-21 °C and H1µM-21 °C groups throughout the reperfusion period. Additionally, the perfusate pO_2_ levels stayed relatively consistent over time for each group (Figure 2A). The H1µM-21 °C group exhibited higher perfusate pH than the other three groups throughout the reperfusion period. Apart from the elevated pH of this group and the high variability at the 1 h timepoint, perfusate pH was maintained within the range of 7.1 to 7.4. Interestingly, perfusate pH decreased over time for each group (Figure 2B). Furthermore, all four groups exhibited comparable increases in lactate levels throughout the reperfusion period (Figure 2C), which contributes to the decrease in perfusate pH described earlier (Figure 2B). None of the differences in perfusate parameter levels were statistically significant.

### 2.2. Subnormothermic Perfusion with Hemopure + 200 nM AP39 at 21 °C Improves Tissue Oxygenation during Blood-Free Preservation

**Figure 3:**
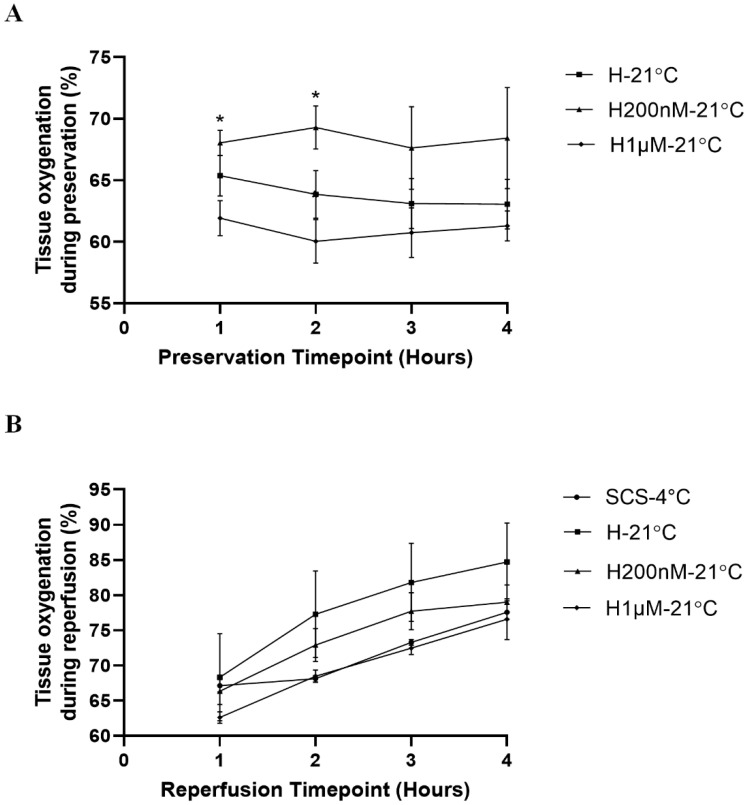
Mean tissue oxygenation during blood-free preservation and reperfusion. (**A**) Tissue oxygenation (%) during 4 h of blood-free preservation with Hemopure (H-21 °C, *n* = 5), Hemopure + 200 nM AP39 (H200nM-21 °C, *n* = 5) or Hemopure + 1 µM AP39 (H1µM-21 °C, *n* = 4). No values were recorded for the static cold storage (SCS-4 °C, *n* = 6) group as the kidneys were on ice. (**B**) Tissue oxygenation (%) during 4 h of blood-free reperfusion with Hemopure and metabolic support at 37 °C. The InSpectra StO2 Spot Check Tissue Perfusion Monitor was unable to detect tissue oxygenation for one pair of kidneys (*n* = 2) in the H1µM-21 °C group. Individual points on each graph represent the mean tissue oxygenation (%) level ± SEM at a specific timepoint for individual kidneys within a single treatment group. Mean ± SEM values are listed in Table 2. After a Geisser–Greenhouse correction, values were compared using repeated measures two-way ANOVA followed by Tukey’s post-hoc test. *, *p* < 0.05 for H200nM-21 °C compared to H1µM-21 °C.

To evaluate the impact of the treatments on organ perfusion, tissue oxygenation was measured hourly during preservation and reperfusion (Figure 3, Table 2). During preservation, the H200nM-21 °C group exhibited higher tissue oxygenation at all timepoints relative to the H-21 °C and H1µM-21 °C groups. Statistically, the H200nM-21 °C group exhibited significantly higher tissue oxygenation than the H1µM-21 °C group at the 1 h and 2 h timepoints during preservation (Figure 3A). During reperfusion, the H-21 °C group exhibited the highest tissue oxygenation, followed by the H200nM-21 °C group. The SCS-4 °C and the H1µM-21 °C groups exhibited similar tissue oxygenation trends and the levels for both groups were lower than the other two groups at the 2 h, 3 h and 4 h timepoints (Figure 3B). Interestingly, the tissue oxygenation levels for each group stayed relatively consistent throughout the preservation period (Figure 3A), while the levels gradually increased for each group throughout the reperfusion period (Figure 3B).

### 2.3. Subnormothermic Perfusion with Hemopure + 200 nM AP39 at 21 °C Improves Kidney Function during Blood-Free Preservation and Reperfusion

**Figure 4:**
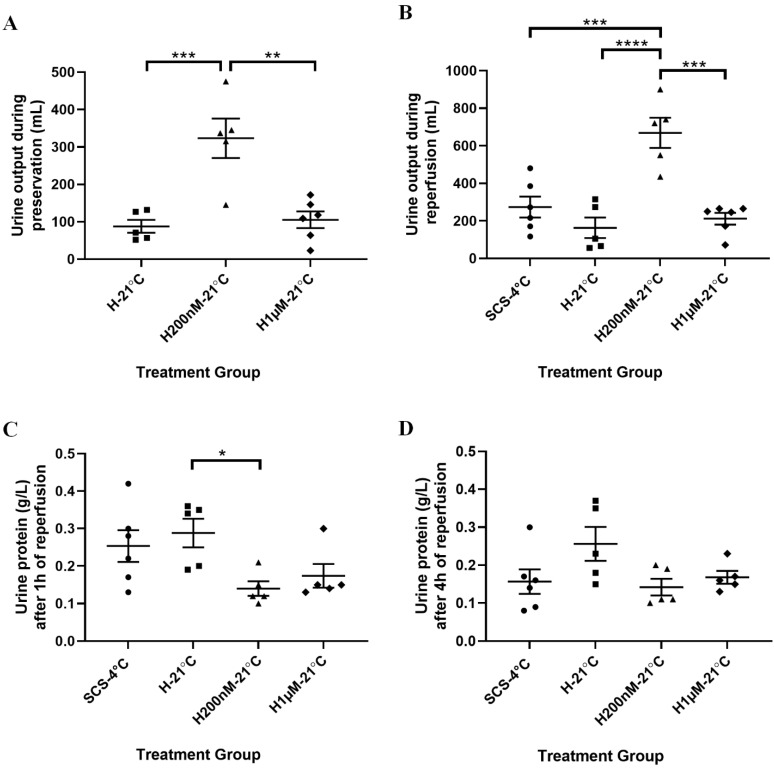
Urine parameters during blood-free preservation and reperfusion. (**A**) Total urine output (mL) during 4 h of blood-free preservation with Hemopure (H-21 °C, *n* = 5), Hemopure + 200 nM AP39 (H200nM-21 °C, *n* = 5) or Hemopure + 1 µM AP39 (H1µM-21 °C, *n* = 6). No values were recorded for the static cold storage (SCS-4 °C, *n* = 6) group as the kidneys were on ice. (**B**) Total urine output (mL) during 4 h of blood-free reperfusion with Hemopure and metabolic support at 37 °C. (**C**) Urine protein (g/L) levels in urine samples collected 1 h after the start of reperfusion. (**D**) Urine protein (g/L) levels in urine samples collected in the final hour (4 h) of reperfusion. An outlier was excluded from the H1µM-21 °C group in (**D**). Individual points on each graph reflect values for individual kidneys and lines represent the mean ± SEM. Values were compared using one-way ANOVA followed by Tukey’s post-hoc test. *, *p* < 0.05. **, *p* < 0.01. ***, *p* < 0.001. ****, *p* < 0.0001.

To evaluate kidney function, urine output was measured during both halves of the experiment and urinalysis was performed (Figure 4). During preservation, the H200nM-21 °C group exhibited significantly higher urine output than the H-21 °C and H1µM-21 °C groups (Figure 4A). This trend continued during reperfusion as the H200nM-21 °C group exhibited significantly higher urine output than the SCS-4 °C, H-21 °C and H1µM-21 °C groups (Figure 4B). Since no urine was collected for the SCS-4 °C group during preservation as the kidneys were on ice, urine samples collected at the 1 h and 4 h timepoints during reperfusion were analyzed to compare all four groups. Samples collected from the H200nM-21 °C group at the 1 h timepoint exhibited lower urine protein levels than all the other groups; however, only the difference between the H-21 °C and the H200nM-21 °C group was statistically significant (Figure 4C). This trend continued as the samples collected from the H200nM-21 °C group at the 4 h timepoint also exhibited lower urine protein levels than all the other groups. However, no statistically significant differences were found between urine protein levels at the 4 h timepoint (Figure 4D).

### 2.4. Subnormothermic Perfusion with Hemopure + 200 nM AP39 at 21 °C Reduces Apoptotic Kidney Injury Following Blood-Free Preservation and Reperfusion

**Figure 5:**
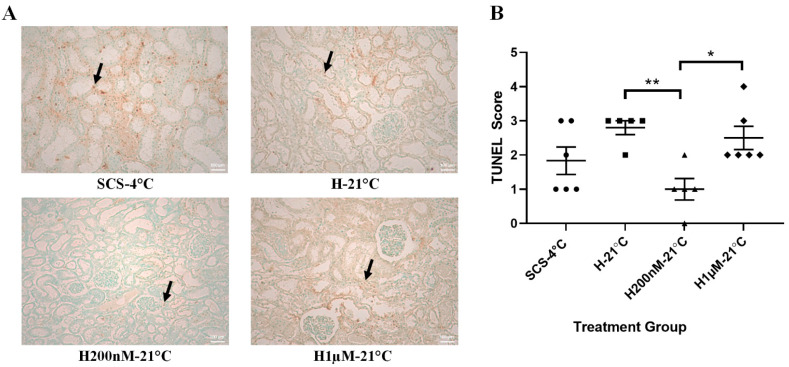
Apoptotic tissue injury following blood-free preservation and reperfusion. (**A**) Representative TUNEL images of formalin-fixed kidney sections after 4 h of blood-free preservation treatment and 4 h of blood-free reperfusion. Images were taken at 10× magnification (scale bar = 100 µm). Black arrows represent apoptotic cells. (**B**) TUNEL scores assigned by a blinded renal pathologist (1 = <11%, 2 = 11–24%, 3 = 25–45%, 4 = 46–75%, 5 = >75%). Each individual data point represents the score assigned to one porcine kidney sample. Lines represent mean ± SEM. Values were compared using one-way ANOVA followed by Tukey’s post-hoc test. *, *p* < 0.05. **, *p* < 0.01. Treatment groups: SCS-4 °C, static cold storage on ice at 4 °C (*n* = 6). H-21 °C, perfusion with Hemopure at 21 °C (*n* = 5). H200nM-21 °C, perfusion with Hemopure + 200 nM AP39 at 21 °C (*n* = 5). H1µM-21 °C, perfusion with Hemopure + 1 µM AP39 at 21 °C (*n* = 6).

**Figure 6:**
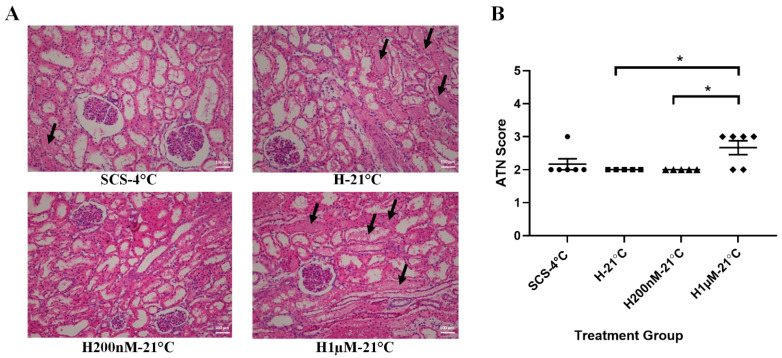
Acute tubular necrosis following blood-free preservation and reperfusion. (**A**) Representative H&E images of formalin-fixed kidney sections after 4 h of blood-free preservation treatment and 4 h of blood-free reperfusion. Images were taken at 10× magnification (scale bar = 100 µm). Black arrows indicate areas of acute tubular injury, including tubular cell sloughing, epithelial flattening and tubular dilation. (**B**) Acute tubular necrosis (ATN) scores assigned by a blinded renal pathologist (1 = <11%, 2 = 11–24%, 3 = 25–45%, 4 = 46–75%, 5 = >75%). Each individual data point represents the score assigned to one porcine kidney sample. Lines represent mean ± SEM. Values were compared using one-way ANOVA followed by Tukey’s post-hoc test. *, *p* < 0.05. Treatment groups: SCS-4 °C, static cold storage on ice at 4 °C (*n* = 6). H-21 °C, perfusion with Hemopure at 21 °C (*n* = 5). H200nM-21 °C, perfusion with Hemopure + 200 nM AP39 at 21 °C (*n* = 5). H1µM-21 °C, perfusion with Hemopure + 1 µM AP39 at 21 °C (*n* = 6).

To evaluate apoptotic tissue injury, kidney sections were stained with TUNEL (Figure 5A) and scored by a blinded renal pathologist (Figure 5B). The H200nM-21 °C group received lower TUNEL scores than all the other groups, which indicates that this group exhibited the lowest apoptotic injury. Statistically, the TUNEL scores of the H200nM-21 °C group were significantly lower than the scores of the H-21 °C and H1µM-21 °C groups. Although the TUNEL scores of the H200nM-21 °C group were also lower than the scores of the SCS-4 °C group, the difference was not statistically significant (Figure 5B). To evaluate acute tubular necrosis (ATN), kidney sections were stained with H&E (Figure 6A) and scored by a blinded renal pathologist (Figure 6B). The SCS-4 °C, H-21 °C and H200nM-21 °C groups received similar ATN scores. However, the ATN scores of the H1µM-21 °C group were significantly higher than the scores of the H-21 °C and H200nM-21 °C groups, which indicates that this group exhibited more severe ATN. Although the ATN scores of the H1µM-21 °C group were also higher than the scores of the SCS-4 °C group, the difference was not statistically significant (Figure 6B).

## 3. Discussion

This study establishes a novel blood-free model of ex vivo kidney preservation and reperfusion using Hemopure, a hemoglobin-based oxygen carrier that serves as a blood substitute. Using this model, we show that subnormothermic perfusion of DCD pig kidneys at 21 °C with AP39-supplemented Hemopure improves graft function and reduces tissue injury compared to SCS and subnormothermic perfusion with Hemopure alone.

The primary finding of this study is that the H200nM-21 °C group displayed significantly improved or comparable outcomes relative to the SCS-4 °C group, which reflects the clinical standard of care for kidney preservation. Importantly, the H200nM-21 °C group exhibited significantly higher urine output than the SCS-4 °C group during reperfusion. The immediacy of post-operative urine output is a critical renal transplant outcome, as it determines whether dialysis is needed to address delayed graft function. In 2018, Hosgood et al. [28] successfully transplanted declined human kidneys after assessing several parameters of renal function, including urine output, during 1 h of ex vivo normothermic perfusion. The five kidneys that were transplanted based on their criteria had higher urine outputs than those that were not transplanted, and only one kidney exhibited delayed graft function. Although additional research is needed to validate their method, their findings suggest that ex vivo urine output can be used to determine whether a kidney is suitable for transplant. While we are yet to evaluate our novel preservation approach using declined human kidneys, the difference in urine output observed in this study has promising implications.

In addition to showing that our novel approach matches the clinical standard of care, this study strengthens the evidence supporting the use of 200 nM AP39 in kidney preservation. We have previously shown that prolonged SCS in UW + 200 nM AP39 improves recipient outcomes in an in vivo model of murine kidney transplantation [11]. Additionally, this dose has shown efficacy in our recent studies on the use of AP39 in subnormothermic kidney preservation [20,27]. From a mechanistic standpoint, our in vitro research has shown that 200–400 nM AP39 preserves mitochondrial membrane potential along with reducing apoptosis and the production of reactive oxygen species [11]. Furthermore, RNA sequencing analysis of DCD pig kidneys preserved with AP39-supplemented blood in our previous study implicated the downregulated expression of pro-apoptotic and hypoxia-response genes as potential mechanisms underlying the protective effects of AP39 [20]. The inclusion of the H1µM-21 °C group in the present study is our first attempt at using a higher dose of AP39 in a mammalian model. The 1 µM dose was chosen based on its efficacy in a frostbite model (unpublished). Seeing that the H1µM-21 °C group exhibited significantly lower urine output and significantly higher tissue injury than the H200nM-21 °C group, this study further supports the use of a low dose (200 nM) AP39. Interestingly, the differences in the outcomes of the H-21 °C and SCS-4 °C groups in the present study do not match expectations and also showed comparable outcomes. A previous study conducted at our center showed that subnormothermic perfusion with Hemopure significantly improved urine output and reduced tissue injury compared to SCS [19]. 

One of the focal points of this study is our novel blood-free model of preservation and reperfusion. While Hemopure has been used for normothermic and subnormothermic kidney preservation before [19,20,26], we are the first to use it consecutively for subnormothermic preservation at 21 °C and normothermic reperfusion at 37 °C. With the efficacy of AP39-suppplemented Hemopure during preservation, we have circumvented a major roadblock to translation—the acquisition of human blood for DCD kidney preservation. Additionally, we used Hemopure for reperfusion to establish a completely blood-free platform for evaluating targeted therapies. While no standard perfusate composition or perfusion protocol exists, the supplements added to the Hemopure/PlasmaLyte mixture in this study align with those used in previous studies reviewed by Elliot et al. [29]. 

On the other hand, our novel perfusion model has several limitations. Although we used Hemopure to establish a blood-free model of preservation and reperfusion, the lack of white blood cells in our perfusate largely excludes the inflammatory component of subnormothermic preservation as well as reperfusion with blood. Additionally, the pigmented nature of Hemopure, due to the hemoglobin, prevented the use of colorimetric and fluorescent assays to detect inflammatory markers in the perfusate. Furthermore, pairs of kidneys were connected to the same circuit and perfused using a shared Hemopure reservoir due to the limitation of having only one organ perfusion pump. Although we collected urine output separately for each kidney, the perfusate parameter readings were less robust, as only one reading was obtained due to the perfusate being shared by pairs of kidneys. Moreover, we were unable to detect perfusate parameters (pO_2_, pH and Lactate) with our iSTAT analysis platform since it was designed for use with blood (not Hemopure) at normothermic temperatures rather than subnormothermic temperatures. Our attempt to use the IDEXX analysis platform to detect perfusate levels during preservation also failed. Moreover, our study lacks a number of controls such as cold or subnormothermic perfusion group without Hemopure. This is because the high cost (CAD 5000/pig experiment) limits the number of groups we could evaluate. Thus, we designed our study around the premise of comparing the outcomes of our novel approach to that of SCS, which is the clinical standard of care, and perfusion with Hemopure alone, which is the control for the effects of temperature and oxygenated perfusion. Lastly, while perfusion was kept at constant pressure by adjusting the flow, we are unable to report flow data. 

As mentioned above, this study advances our previous findings that showed the efficacy of preserving DCD pig kidneys using subnormothermic perfusion at 21 °C with AP39-supplemented blood [20]. There is some overlap in the strengths and limitations of both studies due to the overlap in methodology. Per our previous study, we induced warm ischemic injury by clamping the renal pedicle. This approach mimics an extreme clinical DCD scenario where no oxygen is supplied to the kidneys due to a complete cessation of blood flow. Thus, the positive outcomes observed would likely be heightened in real clinical DCD scenarios, where there is a gradual reduction in blood flow as the donor’s heart stops pumping blood. Additionally, the 4 h duration of the preservation and reperfusion periods is relatively short. However, this was deemed appropriate for establishing a novel model and for facilitating comparisons to our previous study. 

While no mechanistic advances were made, our methodology has improved with the addition of new approaches to evaluate urine protein levels and tissue oxygenation in real time. It is important to note that whereas this study reports a novel approach for subnormothermic kidney preservation with H_2_S-supplemented blood substitute, future ex vivo perfusion studies using declined human kidneys and longer perfusion times are needed to support our findings. Additionally, more in vitro research is required to establish the exact mechanisms underlying the protective effects of AP39. Furthermore, research with clinically approved H_2_S donors and in vivo models of renal transplantation is also needed to facilitate the clinical translation of our novel approach.

In conclusion, this study demonstrates that subnormothermic perfusion at 21 °C with AP39-supplemented Hemopure improves ex vivo DCD porcine renal graft outcomes. Our findings contribute to the expanding body of literature that supports the use of H_2_S and subnormothermic preservation to improve kidney outcomes following transplantation. Additionally, we have established a novel blood-free model of ex vivo kidney preservation and reperfusion that will be useful for evaluating other therapeutics, such as other gasotransmitters and gene therapies. 

## 4. Materials and Methods

### 4.1. Animal Care and Surgery

Yorkshire pigs (60–70 kg), purchased from a regional farm, were tranquilized and routinely prepped for surgery. A midline incision was used to expose the kidneys. Following intravenous infusion of 10,000 U of heparin, the renal pedicles were clamped in situ for 30 min to induce warm ischemia and mimic DCD injury. The complete cessation of renal blood flow replicates an extreme clinical DCD scenario where no oxygen is being supplied to the kidneys. This approach has been used in many other studies within the field and previous studies by our center. During the clamping period, the ureters and arteries were cannulated to facilitate ex vivo perfusion and urine collection. Subsequently, both kidneys were nephrectomized and the donor animal was euthanized. Surgeries were performed by transplant fellows at University Hospital, London, Canada. All procedures were approved by the University of Western Ontario’s Animal Use Committee (Animal Use Protocol 2018-090) on 28 March 2019.

### 4.2. Ex Vivo Perfusion Setup

**Figure 7:**
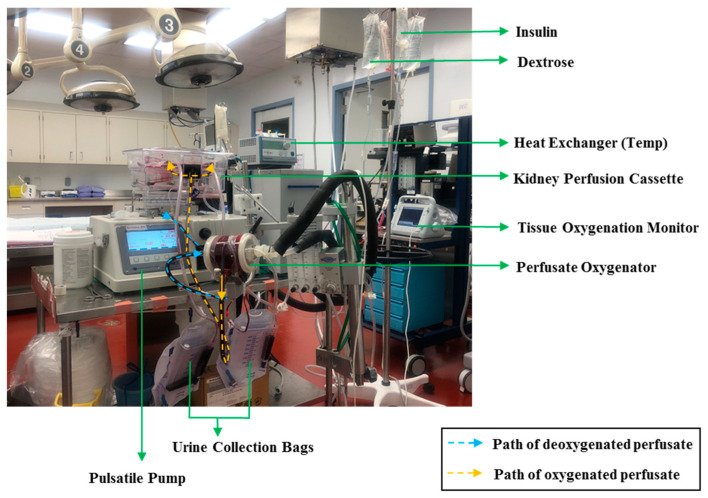
The ex vivo pulsatile perfusion setup used for blood-free preservation and reperfusion. A pair of kidneys, placed in the perfusion cassette, are receiving externally oxygenated perfusate through their arteries. Their ureters are connected to urine collection bags to measure and collect each kidney’s urine output individually. A water-based heat exchanger is connected to the perfusate oxygenator to control the temperature of the perfusate. The image specifically reflects the setup for reperfusion due to the presence of dextrose and insulin drips.

The ex vivo perfusion setup used in this study is identical to the setup used in previous studies [18,19,20] by our center (Figure 7). Mean perfusion pressure was maintained at 60 mmHg through adjusting the flow of the perfusate. Fresh perfusate (1 L) was prepared for preservation and reperfusion by mixing 250 mL of Hemopure (generously provided by HbO2 Therapeutics, Souderton, PA, USA) with 750 mL of PlasmaLyte solution (Baxter International Inc., Deerfield, IL, USA). The perfusate was supplemented with the following: heparin (5000 U), sodium bicarbonate (8.4%, 10 mL) and Ancef (1 g). Pairs of kidneys were perfused together due to having a single pulsatile pump. However, each kidney was considered as one replicate because its urine output was collected individually.

### 4.3. Blood-Free Preservation Treatments

**Figure 8:**
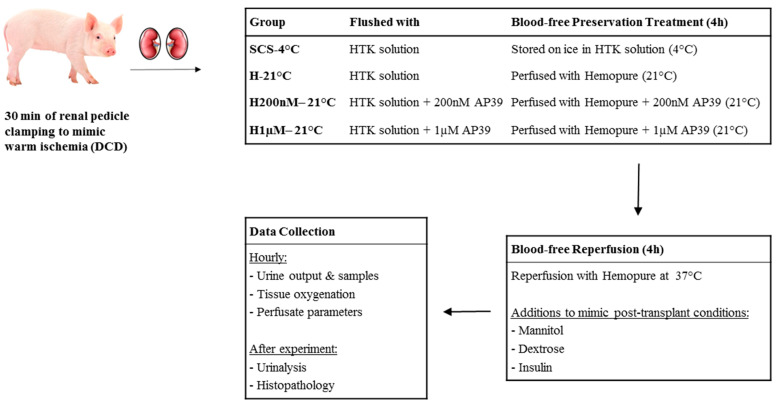
Summary of the preservation treatment groups and methodology.

Pairs of kidneys were assigned to one of four treatment groups (Figure 8). The first group of kidneys were flushed with and stored in Histidine-Tryptophan-Ketoglutarate (HTK) solution (Custodiol^®^, USA) on ice for 4 h (SCS-4 °C), which reflects the clinical standard of care. The second group of kidneys were flushed with HTK solution and treated to 4 h of subnormothermic perfusion at 21 °C with Hemopure (H-21 °C). The third group of kidneys were flushed with HTK solution + 200 nM AP39 and treated to 4 h of subnormothermic perfusion at 21 °C with Hemopure + 200 nM AP39 (H200nM-21 °C). The fourth group of kidneys were flushed with HTK solution + 1 µM AP39 and treated to 4 h of subnormothermic perfusion at 21 °C with Hemopure + 1 µM AP39 (H1µM-21 °C). For the three preservation treatments involving subnormothermic perfusion, urine output was recorded hourly and the volume loss was replaced with the addition of PlasmaLyte. Additionally, tissue oxygenation was measured hourly using the InSpectra StO_2_ Spot Check Tissue Perfusion Monitor (Hutchinson Technology, Hutchinson, MN, USA).

### 4.4. Blood-Free Reperfusion Protocol

Following 4 h of preservation, kidneys were reperfused for 4 h using our novel blood-free reperfusion model (Figure 2). Due to budget constraints, the same perfusion cassettes were used throughout preservation and reperfusion. To prevent the mixing of the perfusates from both halves of the experiment, the perfusion circuit was drained and flushed with 2 L of saline between preservation and reperfusion. After the saline flush, 1 L of fresh Hemopure/PlasmaLyte solution was added for reperfusion and the temperature was set to 37 °C. At the start of reperfusion, 4 g of mannitol was added to the perfusate to mimic the post-operative administration of a diuretic to renal transplant recipients. Additionally, we implemented 5% dextrose and insulin drips to provide metabolic support to the kidneys, maintaining a perfusate glucose concentration of ~150 mg/dL.

During reperfusion, urine samples were collected, and urine output was recorded hourly. The volume lost was replaced with the addition of PlasmaLyte. Tissue oxygenation was measured hourly using the InSpectra StO_2_ Spot Check Tissue Perfusion Monitor (Hutchinson Technology, Hutchinson, MN, USA). Additionally, perfusate parameters (pH, pO_2_, and lactate) were measured using the iSTAT Handheld Blood Analyzer (Abbott Laboratories, Chicago, IL, USA) to allow for relative comparison between groups. Sodium bicarbonate was injected as needed to adjust perfusate pH. After 4 h of reperfusion, kidney sections (cortex and medulla) were cut and stored in formalin for histopathological analyses.

### 4.5. Hydrogen Sulfide Donor Molecule AP39

Hydrogen sulfide donor molecule, AP39, synthesized in-house by Prof. Whiteman [30], was dissolved in dimethyl sulfoxide to achieve a 1 mM stock concentration. To achieve a treatment concentration of 200 nM AP39, 200 µL of the stock was added to 1 L of preservation solution and perfusate. Similarly, for a concentration of 1 µM AP39, 1 mL of the stock was added to 1 L of preservation solution and perfusate. The doses were chosen based on previous studies by our group [20].

### 4.6. Urinalysis

Most of the urine samples collected were heavily pigmented, due to the presence of hemoglobin from the Hemopure, which prevented the use of conventional urinalysis methods. A 1:3 dilution of urine in Hemoglobind (Biotech Support Group, Monmouth Junction, NJ, USA) allowed us to obtain clearer urine samples after 10 min of vigorous shaking and centrifugation at 12,000× *g*. Urine protein and creatinine levels were analyzed using the IDEXX Urine Analyzer (IDEXX Laboratories, Westbook, ME, USA), but creatinine values remained undetectable.

### 4.7. Histopathology Imaging and Scoring

Formalin-fixed kidney sections, including cortex and medulla, were embedded in paraffin and mounted onto microscope slides. The sections were stained with Terminal deoxynucleotidyl transferase dUTP nick end labeling (TUNEL) and Hematoxylin and Eosin (H&E) to determine the level of apoptosis and acute tubular necrosis, respectively. TUNEL and H&E imaging was done using the Nikon Instruments Eclipse 90i digital microscope at 10× magnification (Nikon Instruments, Melville, NY, USA). Both sets of slides were scored by a blinded renal pathologist as per the following scheme: 1 = <11%, 2 = 11–24%, 3 = 25–45%, 4 = 46–75%, 5 = >75%.

### 4.8. Statistical Analyses

GraphPad Prism v9.0 (GraphPad Software, San Diego, CA, USA) was used to create graphs and conduct statistical analyses. SEM is graphed as it accounts for the impact of varying *n* values in certain groups. One-way or two-way ANOVA followed by Tukey’s post-hoc test was used for comparisons of three or more experimental groups. Statistical significance was accepted at *p* < 0.05.

## 5. Patents

The data reported in this study are a part of a US patent application (serial no. 17/127,965 entitled “Method and Compositions for Protecting Tissue) that is pending approval. Additionally, Prof. Matthew Whiteman and the University of Exeter have intellectual property (patent filings) related to hydrogen sulfide delivery molecules and their therapeutic use.

## Figures and Tables

**Table 1:** Mean perfusate parameters ± SEM during blood-free preservation and reperfusion.

**pO_2_ (mmHg)**				
	**Reperfusion Timepoint**			
**Group (*n*)**	**1 h**	**2 h**	**3 h**	**4 h**
SCS-4 °C (*n* = 3)	227.00 ± 6.43	231.00 ± 15.04	231.00 ± 9.644	221.33 ± 9.493
H-21 °C (*n* = 3)	215.66 ± 3.71	221.33 ± 9.59	224.33 ± 6.17	221.33 ± 3.28
H200nM-21 °C (*n* = 3)	242.66 ± 14.19	242.66 ± 18.97	238.66 ± 5.78	241.00 ± 15.50
H1µM-21 °C (*n* = 2)	213.00 ± 13.00	214.00 ± 13.00	215.50 ± 5.50	212.00 ± 8.00
**pH**				
	**Reperfusion Timepoint**			
**Group (*n*)**	**1 h**	**2 h**	**3 h**	**4 h**
SCS-4 °C (*n* = 3)	7.40 ± 0.11	7.27 ± 0.09	7.17 ± 0.08	7.05 ± 0.09
H-21 °C (*n* = 3)	7.67 ± 0.13	7.35 ± 0.13	7.49 ± 0.12	7.38 ± 0.10
H200nM-21 °C (*n* = 3)	7.17 ± 0.04	7.29 ± 0.23	7.24 ± 0.11	7.13 ± 0.03
H1µM-21 °C (*n* = 2)	7.84 ± 0.10	7.71 ± 0.09	7.56 ± 0.08	7.44 ± 0.05
**Lactate (mmol/L)**				
	**Reperfusion Timepoint**			
**Group (*n*)**	**1 h**	**2 h**	**3 h**	**4 h**
SCS-4 °C (*n* = 3)	4.39 ± 0.59	6.51 ± 0.84	8.22 ± 0.69	9.54 ± 0.68
H-21 °C (*n* = 3)	3.34 ± 0.35	4.84 ± 0.81	6.49 ± 0.99	8.35 ± 1.36
H200nM-21 °C (*n* = 3)	3.78 ± 0.12	5.73 ± 0.12	7.023 ± 0.68	9.64 ± 1.02
H1µM-21 °C (*n* = 2)	2.90 ± 0.90	5.79 ± 0.79	8.29 ± 0.29	10.69 ± 0.48

Note: SCS-4 °C, static cold storage on ice at 4 °C. H-21 °C, perfusion with Hemopure at 21 °C. H200nM-21 °C, perfusion with Hemopure + 200 nM AP39 at 21 °C. H1µM-21 °C, perfusion with Hemopure + 1 µM AP39 at 21 °C.

**Table 2:** Mean tissue oxygenation ± SEM (%) during blood-free preservation and reperfusion.

	Mean Tissue Oxygenation (%) ± SEM (%)			
	**Preservation Timepoint**			
**Group (*n*)**	**1 h**	**2 h**	**3 h**	**4 h**
SCS-4 °C (*n* = 6)	No data collected while kidneys were on ice			
H-21 °C (*n* = 5)	65.37 ± 1.65	63.86 ± 1.94	63.11 ± 2.04	63.06 ± 2.02
H200nM-21 °C (*n* = 5)	**68.04 ± 1.02**	**69.29 ± 1.76**	67.62 ± 3.36	68.43 ± 4.10
H1µM-21 °C (*n* = 4)	**61.93 ± 1.42**	**60.04 ± 1.78**	60.75 ± 2.02	61.29 ± 1.21
	**Reperfusion Timepoint**			
**Group (*n*)**	**1 h**	**2 h**	**3 h**	**4 h**
SCS-4 °C (*n* = 6)	67.12 ± 0.88	68.12 ± 0.47	73.29 ± 0.40	77.57 ± 0.24
H-21 °C (*n* = 5)	68.31 ± 6.20	77.29 ± 6.15	81.80 ± 5.55	84.71 ± 5.52
H200nM-21 °C (*n* = 5)	66.35 ± 1.91	72.90 ± 2.34	77.73 ± 2.62	79.00 ± 2.44
H1µM-21 °C (*n* = 4)	62.61 ± 0.82	68.46 ± 0.89	72.46 ± 0.91	76.57 ± 2.91

Note: Bolded and highlighted values in each column were significantly different relative to each other (*p* < 0.05) as per repeated measures two-way ANOVA and Tukey’s post-hoc test after a Geisser–Greenhouse correction. SCS-4 °C, static cold storage on ice at 4 °C. H-21 °C, perfusion with Hemopure at 21 °C. H200nM-21 °C, perfusion with Hemopure + 200 nM AP39 at 21 °C. H1µM-21 °C, perfusion with Hemopure + 1 µM AP39 at 21 °C.

## Data Availability

The data presented in this study are available on request from the corresponding author.
